# Pitfalls of endoscopic ultrasound-guided gallbladder drainage using a lumen-apposing metal stent: impact of the intraluminal water-filling technique

**DOI:** 10.1055/a-2705-2435

**Published:** 2025-10-02

**Authors:** Takeshi Ogura, Junichi Nakamura, Takafumi Kanadani, Kimi Bessho, Hiroki Nishikawa

**Affiliations:** 1 38588 Pancreatobiliary Advanced Medical Center, Osaka Medical and Pharmaceutical University Hospital, Osaka, Japan; 2 38588 Endoscopy Center, Osaka Medical and Pharmaceutical University Hospital, Osaka, Japan; 3 13010 2nd Department of Internal Medicine, Osaka Medical and Pharmaceutical University, Osaka, Japan

Endoscopic ultrasound-guided gallbladder drainage (EUS-GBD) using a lumen-apposing metal stent (LAMS) is now widely attempted in patients for whom surgical treatment is contraindicated 1 2 . When an electrocautery-enhanced LAMS (Hot AXIOS, Boston Scientific) is deployed, the distance between the gallbladder and the lumen should be <1 cm to prevent stent misdeployment. During maneuvering of the echoendoscope to identify the gallbladder, the intestinal wall may sometimes be folded and, as a result, the distance between the gallbladder and the lumen may be measured as being further away than it actually is. This finding may result in a false contraindication to EUS-GBD using a LAMS. In addition, if EUS-GBD is performed in this situation, double mucosal puncture can occur. To prevent these errors, the water-filling technique may be useful. Here, we describe some technical tips for EUS-GBD using a LAMS combined with the water-filling technique.

First, the echoendoscope was inserted into the duodenum, and the gallbladder was identified. The distance between the gallbladder wall and the duodenum was found to be 1.5 cm ( Fig. 1 **a** ). In this situation, because the distance between the positions of the distal and proximal flanges was not <1 cm, EUS-GBD using a LAMS may not be indicated. In addition, because the double mucosal sign could be observed, double mucosal puncture might occur. To prevent these errors, approximately 40 mL of saline was injected into the duodenum via the working channel of the echoendoscope ( Fig. 1 **b** ), and an echo-free space was obtained. The distance between the gallbladder and the duodenum was then found to be extremely small. Subsequently, the LAMS delivery system was inserted up to the duodenal wall ( Fig. 1 **c** ) and was then inserted into the gallbladder using the electrocautery system ( Fig. 1 **d** ). After the distal flange had been opened, the stent delivery system was pulled back, and stent release was completed using the intrascope channel re-release technique ( Fig. 1 **e** ). Stent deployment was thereby successfully performed without any adverse events ( Video 1 ).

**Fig. 1:**
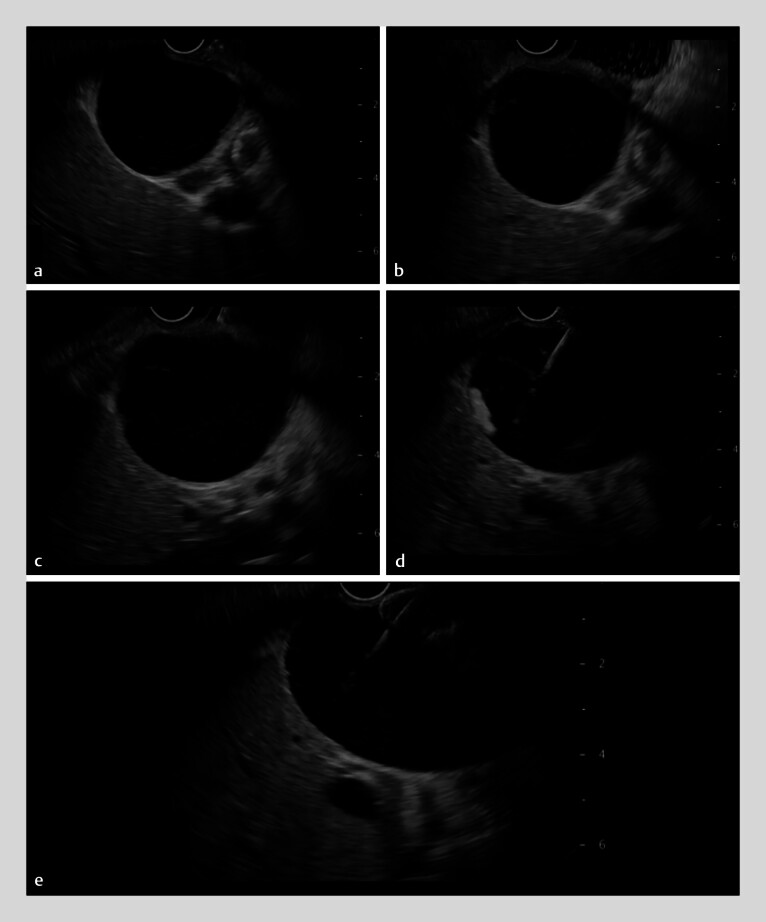
Endoscopic ultrasound images showing: **a** the initial distance between the gallbladder wall and the duodenum being measured as 1.5 cm; **b** approximately 40 mL of saline injected into the duodenum via the working channel of the echoendoscope to create an echo-free space; **c** the lumen-apposing metal stent (LAMS) delivery system inserted up to the duodenal wall; **d** the LAMS delivery system inserted into the gallbladder using the electrocautery system; **e** completion of stent release using the intrascope channel re-release technique.

Endoscopic ultrasound-guided gallbladder drainage is performed with a lumen-apposing metal stent using the intraluminal water-filling technique.Video 1

In conclusion, EUS-GBD using a LAMS combined with the water-filling technique can prevent both a missed indication for EUS-GBD and potential double mucosal puncture.

Endoscopy_UCTN_Code_TTT_1AS_2AD

## Notes

Endoscopy E-Videos https://eref.thieme.de/e-videos
*E-Videos* is an open access online section of the journal *Endoscopy* , reporting on interesting cases and new techniques in gastroenterological endoscopy. All papers include a high-quality video and are published with a Creative Commons CC-BY license. Endoscopy E-Videos qualify for HINARI discounts and waivers and eligibility is automatically checked during the submission process. We grant 100% waivers to articles whose corresponding authors are based in Group A countries and 50% waivers to those who are based in Group B countries as classified by Research4Life (see: https://www.research4life.org/access/eligibility/ ). This section has its own submission website at https://mc.manuscriptcentral.com/e-videos .
